# Expression alteration of serum exosomal circular RNAs in obstructive sleep apnea patients with acute myocardial infarction

**DOI:** 10.1186/s12920-023-01464-4

**Published:** 2023-03-09

**Authors:** Jie-feng Huang, Ning-Fang Lian, Guo-Fu Lin, Han-Sheng Xie, Bi-Ying Wang, Gong-Ping Chen, Qi-Chang Lin

**Affiliations:** 1 Fujian Provincial Sleep-Disordered Breathing Clinic Center, Institute of Respiratory Disease, Fujian Medical University, No 20, Chazhong Road, Taijiang District, Fuzhou, 350005 Fujian Province People’s Republic of China; 2 Department of Respiratory and Critical Care Medicine, First Affiliated Hospital of Fujian Medical University, Fuzhou, People’s Republic of China; 3 Department of Pulmonary and Critical Care Medicine, National Regional Medical Center, Binhai Campus of the First Affiliated Hospital, Fujian Medical University, Fuzhou, People’s Republic of China

**Keywords:** Circular RNAs, Exosomes, Obstructive sleep apnea, Acute myocardial infarction, Diagnostic biomarker

## Abstract

**Purpose:**

Circular RNAs (circRNAs) are recently identified as a class of non-coding RNAs that participate in the incidence of acute myocardial infarction (AMI). However, circRNAs expression pattern in obstructive sleep apnea (OSA) with AMI remains unknown. The aim was to investigate circRNAs expression alteration in serum exosomes derived from OSA patients with AMI.

**Methods:**

The serum exosomal circRNAs profile of three healthy subjects, three OSA without AMI and three OSA with AMI were analyzed using high-throughput sequencing. Bioinformatic analyses were carried out to assess potential core circRNAs and functional analyses were conducted to study biological functions.

**Results:**

Compared to healthy subjects, there were 5225 upregulated and 5798 downregulated circRNAs in exosomes from OSA with AMI patients. And our study also identified 5210 upregulated and 5813 downregulated circRNAs in OSA with AMI patients compared to OSA without AMI. The differential expression of 2 circRNAs (hsa_circRNA_101147, hsa_circRNA_101561) between healthy subjects and OSA without AMI, and 4 circRNAs (hsa_circRNA_101328, hsa_circRNA_104172, hsa_circRNA_104640, hsa_circRNA_104642) between healthy subjects and OSA with AMI were confirmed by qRT-PCR. In addition, we demonstrated that miR-29a-3p targeted hsa_circRNA_104642 directly.

**Conclusions:**

This study demonstrated that there were a number of dysregulated circRNAs in exosomes from OSA with AMI patients, which might be effectively served as a promising diagnostic biomarker and therapeutic targets.

## Introduction

Obstructive sleep apnea (OSA) is characterized by upper airway obstruction causing a cessation or induction of airflow and leading to recurrent intermittent hypoxia and sympathetic activation, which contributes to the onset and progression of cardiac and vascular damage [1–3]. Masaracchia et al. [4] studied a cohort of 128,932 patients between 2010–2015 and found that OSA was associated with a 4.95 (95% CI, 1.81 to 13.5) times greater odds of acute myocardial infarction (AMI). Furthermore, a study reported that the peak occurrence of AMI was between 12 and 6 AM in patients with OSA, which is not accordance with that in general population whose peak occurrence is between 6 AM and 12 PM [5].

Circular RNAs (circRNAs) are a novel class of non-coding RNAs showing no 5′-3′ polarity or polyadenylation, which are more stable than their linear counterparts in contrast to linear RNAs [6]. With advances in high-throughput deep RNA sequencing (RNA-seq) technology, increasing numbers of circRNAs have been screened and involved in regulating the expression of genes in many diseases, including cancer, neurological disorders and cardiovascular disease [7–9]. It has been reported that circRNAs were associated with AMI-related apoptosis [10]. Most circRNAs are located in the cytoplasm to increase the possibility of their early release upon tissue damage [11]. A study conducted by Schulte et al. [12] found that circRNAs in cardiac tissue were high abundance and might serve as biomarkers for AMI. However, cardiac circRNAs were poorly detected in plasma or serum. Recent studies discovered that circRNAs were enriched and stable in exosomes [13, 14]. Exosomes are present in various types of cells under normal or pathological conditions and found in almost all eukaryotic fluids, including blood, and urine, and in the culture medium of cells, which are involved in cell-to-cell signaling and may influence processes in normal cells by merging with and releasing their contents into cells that are distant from their cell of origin [15, 16]. It is speculated that exosomes might act as potential biomarkers for diagnosis of human diseases because they contain a variety of protein and RNA species that can modulate the behavior of recipient cells [17, 18]. Until now, no research reports on serum exosomal circRNAs from OSA with AMI. Therefore, we speculated that circRNAs in exosomes might play an important role in regulating gene expression and regulate specific signaling pathways in OSA patients with AMI.

The objective of this study was to explore expression profiles of serum exosomal circRNAs of three healthy subjects, three OSA without AMI and three OSA with AMI using microarray technology and analyzed biological functions of differently expressed circRNAs by bioinformatics analysis, which may contribute to provide new insights into cardiovascular diseases caused by OSA.

## Materials and methods

### Subjects and protocols

9 subjects were enrolled in the study. They were classified into three groups: three healthy subjects (healthy group), three OSA without AMI and three OSA with AMI. The main symptoms of three AMI patients were chest pain or chest tightness, with dynamic alterations of electrocardiogram and dynamic changes of myocardial injury markers, and confirmed by coronary angiography [19]. The three AMI patients were all diagnosed as ST-segment elevation myocardial infarction (STEMI) with ST-segment elevation in electrocardiograms [19]. Three healthy subjects and three OSA without AMI underwent polysomnography in our sleep center after their admission, and OSA with AMI underwent polysomnography on the day of diagnosis of AMI in the department of cardiology by the same PSG technician. The study was also approved by the Ethics Committee of the First Affiliated Hospital of Fujian Medical University (NO: 2019–048) and written informed consent was obtained from all participants prior to the study. All methods were carried out in accordance with relevant guidelines and regulations. The clinical and demographic characteristics of the study participant are shown in Table 1.

**Table 1:** The clinical information of the patients included in the study

Sample ID	Sex	Age (years)	Diagnosis	AHI	LaSO_2_ (%)	History of HP	History of DM	BMI (kg/m^2^)
A1	F	61	OSA with AMI	35.2	45	yes	not	27.61
A2	F	49	OSA with AMI	40.2	65	yes	not	25.12
A3	F	51	OSA with AMI	43.1	82	yes	not	29.23
B1	F	65	OSA without AMI	42.1	73	not	not	25.56
B2	F	31	OSA without AMI	81.0	33	yes	not	32.53
B3	F	68	OSA without AMI	64.4	85	not	not	28.76
C1	F	35	Healthy subject	3.1	93	not	not	25.32
C2	M	31	Healthy subject	2.5	94	not	not	23.18
C3	F	50	Healthy subject	2.7	95	not	not	25.41

*OSA* obstructive sleep apnea, *AMI* acute myocardial infarction, *F* female, *M* male; *AHI* apnea–hypopnea index, *LaSO*_*2*_ lowest oxygen saturation; *HP* hypertension, *DM* Diabetes Mellitus, *BMI* Body Mass Index

### Samples collection, isolation and characterization of exosomes

Peripheral venous blood samples were obtained in the morning after performing overnight PSG (PSG was conducted after the coronary angiography surgery in patients with AMI). All of blood samples (8 mL from peripheral venous blood) were collected using a conventional blood collection tube (usually a red head), and stored at room temperature for 2 h until a significant clot is produced. After centrifuged at 1200 g for 10 min at 4 °C and 1800 g for 10 min at 4 °C, the supernatants were collected and frozen at -80 °C until use. As ultracentrifugation can accurately and repeatedly obtain exosomes while minimizing co-purification of protein aggregates and other membrane particles, in our study exosomes were isolated by ultracentrifugation and the quality was determined with an electron microscope [20].

### RNA library construction and circRNA sequencing

Total RNA was extracted from exosomes using a standard TRIzol reagent according to the manufacturer’s instructions. RNA quantification and quality assurance were determined with NanoDrop ND‐1000 by measuring the O.D. A260/A280 ratio and the O.D. A260/A230 ratio with ultraviolet spectrophotometry. The extraction of RNA and analysis of circRNA expression profiles were performed according to methods previously reported [9]. Based on the Arraystar’s standard protocols, we firstly enriched circRNAs from total RNA by depleting the linear RNA with Rnase R (Epicentre, Inc.). Then, the enriched and digested circRNAs were amplified and transcribed into fluorescent cRNA utilizing a random priming method (Arraystar Super RNA Labeling Kit; Arraystar). The labeled cRNAs were purified using an RNeasy Mini Kit (Qiagen). NanoDrop ND-1000 was used to measure the concentration and specific activity of the labeled cRNAs (pmol Cy3/μg cRNA). Finally, hybridization solution was placed in a gasket slide, which was then assembled to the circRNA expression microarray slide. The circRNA expression microarray slides were incubated for 17 h at 65 °C in an Agilent Hybridization Oven, then washed, fixed and scanned using the Agilent Scanner G2505C.

Agilent Feature Extraction software (version 11.0.1.1) was used to analyze acquired array images. Differentially expressed circRNAs between two groups were discovered through fold change filtering. Hierarchical clustering was conducted to display the distinct circRNA expression profiling among samples. Data were extracted from images provided by the scanner using Agilent Feature Extraction software for raw data extraction. Quantile normalization of raw data and subsequent data processing were carried out using the R software package.

### Pathway analysis of circRNA gene symbols

To speculate about the potential function of circRNAs, Gene Ontology project (GO) and a Kyoto Encyclopedia of Genes and Genomes (KEGG) pathway were performed by GOATOOLS and molecular function, cell components, and biological processes were included. The *p*-value produced by top GO denoted the significance of GO terms enrichment in the DE genes, and the *p*-value in KEGG denoted the significance of the pathway correlated to the conditions.

### Quantitative real-time PCR (qRT-PCR)

qRT-PCR was performed to confirm the accuracy of the microarray data. Total RNA was treated with Rnase R (Epicentre, Inc.) and then converted to cDNA using a Prime Script RT Reagent Kit (Applied Biosystems, CA, USA). qRT-PCR was conducted by using the Power SYBR Green PCR Master Mix (Applied Biosystems, CA, USA) on the Applied Biosystems 7500 RT-PCR System (Applied Biosystems). The selected circRNAs were normalized to β-actin and the operations were repeated three times. The relative expression of the gene was analyzed by the 2-ΔΔCt method. The primers were listed in Table 2.

**Table 2:** Primers used for qRT- PCR

Primer name	Sequence (5′–3′)	Tm (°C)	Lengths (bp)
β-actin	F:5’GTGGCCGAGGACTTTGATTG3’ R:5’CCTGTAACAACGCATCTCATATT3’	60	73
circRNA_101147	F:5’CCAAGACATATAGAGCAGTTCCAAG3’ R:5’ AATAGGCATGGCAACAGCTTC 3’	60	152
circRNA_101561	F:5’ ACCAGAGAAGAAAGGAGACTCAC 3’ R:5’ TGGATCAGCAGGGCATAAT 3’	60	118
circRNA_101328	F:5’ GGACGGCGTCACCAACCTA 3’ R:5’ GCACGCATTCTTTCTGGACAT 3’	60	150
circRNA_104172	F:5’ TCATCATCTTGAATTTAGTGAAGAG 3’ R:5’ CGATATTTCGTTTCTGCATTATT 3’	60	67
circRNA_104640	F:5’ TCTGTCCCACCCATCCCATCA 3’ R:5’ TCCAAAGAGCATCCCTGCAAAAG 3’	60	171
circRNA_104642	F:5’ ATGCAAAAGGAAATCTGATAAGA 3’ R:5’ CCTGGACCCATAAAAATAGTATG 3’	60	115

### Annotation for circRNA/miRNA interaction and ceRNA

To facilitate researcher’s study, the circRNA/miRNA interaction was predicted with Arraystar's home-made miRNA target prediction software, whose principles are based on TargetScan&miRanda, and the differentially expressed circRNAs within all the comparisons were annotated in detail with the circRNA/miRNA interaction information. Through merging the common targeted miRNAs, we constructed systematic analysis of ceRNA network by using Cytoscape.

### Dual-luciferase reporter assay

Dual-luciferase reporter assays were used to detect the binding interactions between circRNA_104642 and miR-29a-3p. 293 T cells were cultured in 24-well plates and co-transfected with pmirGLO vector (Promega, China) carrying either wild or mutated circRNA_104642 3’UTR sequences together with miR-29a-3p mimics or mimics NC. After transfecting 48 h, firefly and renilla luciferase activities were measured using the dual-luciferase reporter assay (Promega, Madison, WI, USA). The luciferase activity was calculated as the ratio of firefly luciferase intensity to renilla luciferase intensity.

### Statistical analysis

When comparing two groups of profile differences, unpaired t test were conducted. To assess the significance of GO terms or pathway identifiers’ enrichment, Fisher’s exact test was applied. The hypergeometric test were utilized to detect the circRNA-mRNA interactions, and Pearson correlation test was used to evaluate the co-expression of the screened circRNA-mRNA interactions. It was considered significantly differentially expressed when the *p*-values was less than 0.05.

## Results and discussion

### Detection of exosomes

**Fig. 1:**
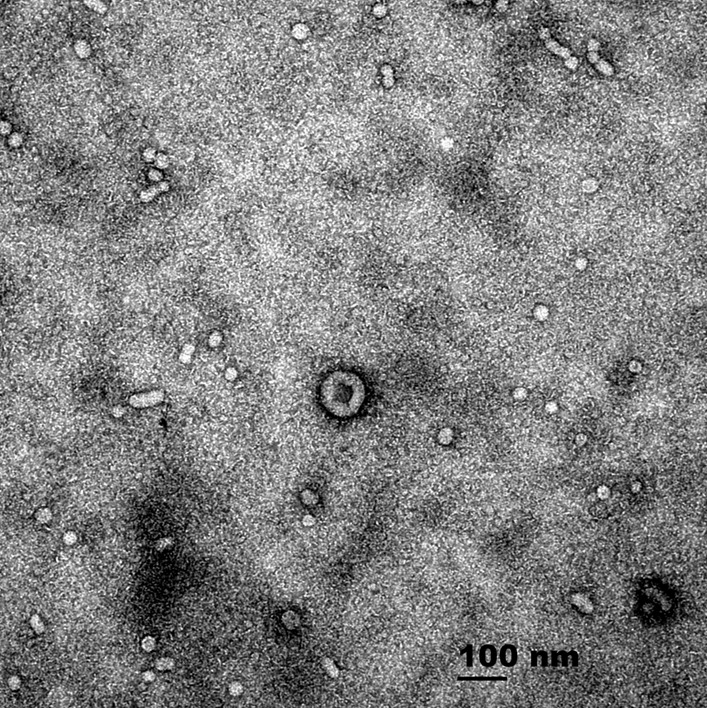
Structure of the exosomes shown by transmission electron microscopy (TEM). The exosome is cystic with double membranes, Scale bar = 100 nm

The clinical and demographic characteristic of this study participant are showed in Table 1. Identification of the exosomes was presented in Fig. 1. Electron microscope showed that the exosomes were about 100 nm in diameter with double membranes.

### Distinguishable expression of circRNAs in exosomes from the serum of patients

**Fig. 2:**
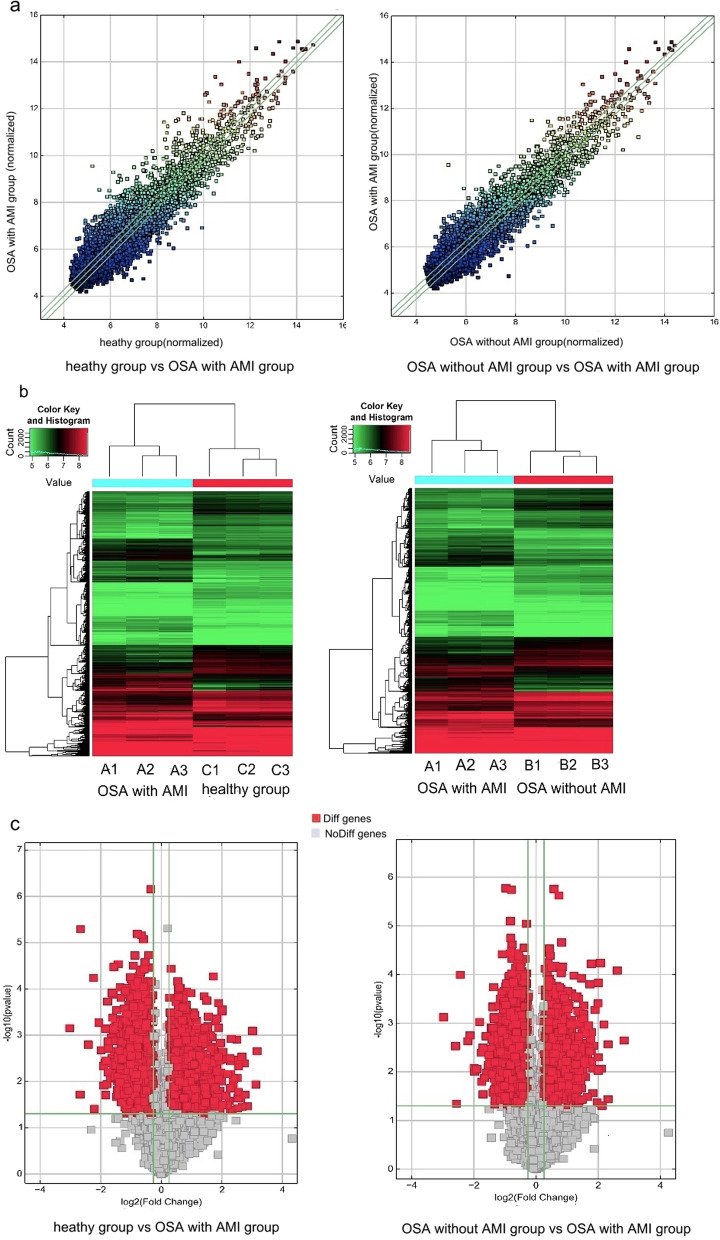
Bioinformatics analysis of circRNAs expression patterns between group of obstructive sleep apnea (OSA) with acute myocardial infarction (AMI), OSA without AMI and healthy subjects using microarray technology. **a** Scatter plot with the mean expression values of each group samples. Raw junction reads for all samples were normalized by total mapped read numbers and were log.^2^ transformed. **b** Heat map evaluation of the distinguishable circRNA expression patterns among the OSA with AMI, OSA without AMI, and healthy group. Each column represents one sample; each row represents one probe set; high and low expression was indicated by the “red” and “green” line, respectively. **c** The volcano graph was created to show significantly dysregulated circRNAs (fold change > 1.5, *p*-value < 0.05)

CircRNA microarray analyses in serum exosomes were performed to identify uniquely differentially expressed circRNAs from healthy group, OSA without AMI group and OSA with AMI group. Compared to healthy subjects, there were 5225 upregulated and 5798 downregulated circRNAs in exosomes from OSA with AMI patients. And our study also identified 5210 upregulated and 5813 downregulated circRNAs in OSA with AMI patients compared to OSA without AMI. Based on the criteria of exhibiting fold change ≥ 1.2 and *p*‐values ≤ 0.05, we identified 14 were up-regulated and 18 were down-regulated in OSA without AMI group compared to the heathy group. Based on the criteria of exhibiting fold change ≥ 2.0 and *p*‐values ≤ 0.05, 350 were up-regulated and 76 were down-regulated, 155 were up-regulated and 259 were down-regulated in OSA with AMI group compared to the healthy group, and 230 were up-regulated and 169 were down-regulated in OSA with AMI group compared to OSA without AMI group. Scatter plot was conducted to value the difference of circRNAs expression between groups (Fig. 2a). Then heat-map clustering was conducted to reveal the distinguishable circRNA expression profile between two groups among the three groups (Fig. 2b). The scatter plot was drawn to analyze the mean expression values of each group samples (Fig. 2c).

### GO analysis

**Fig. 3:**
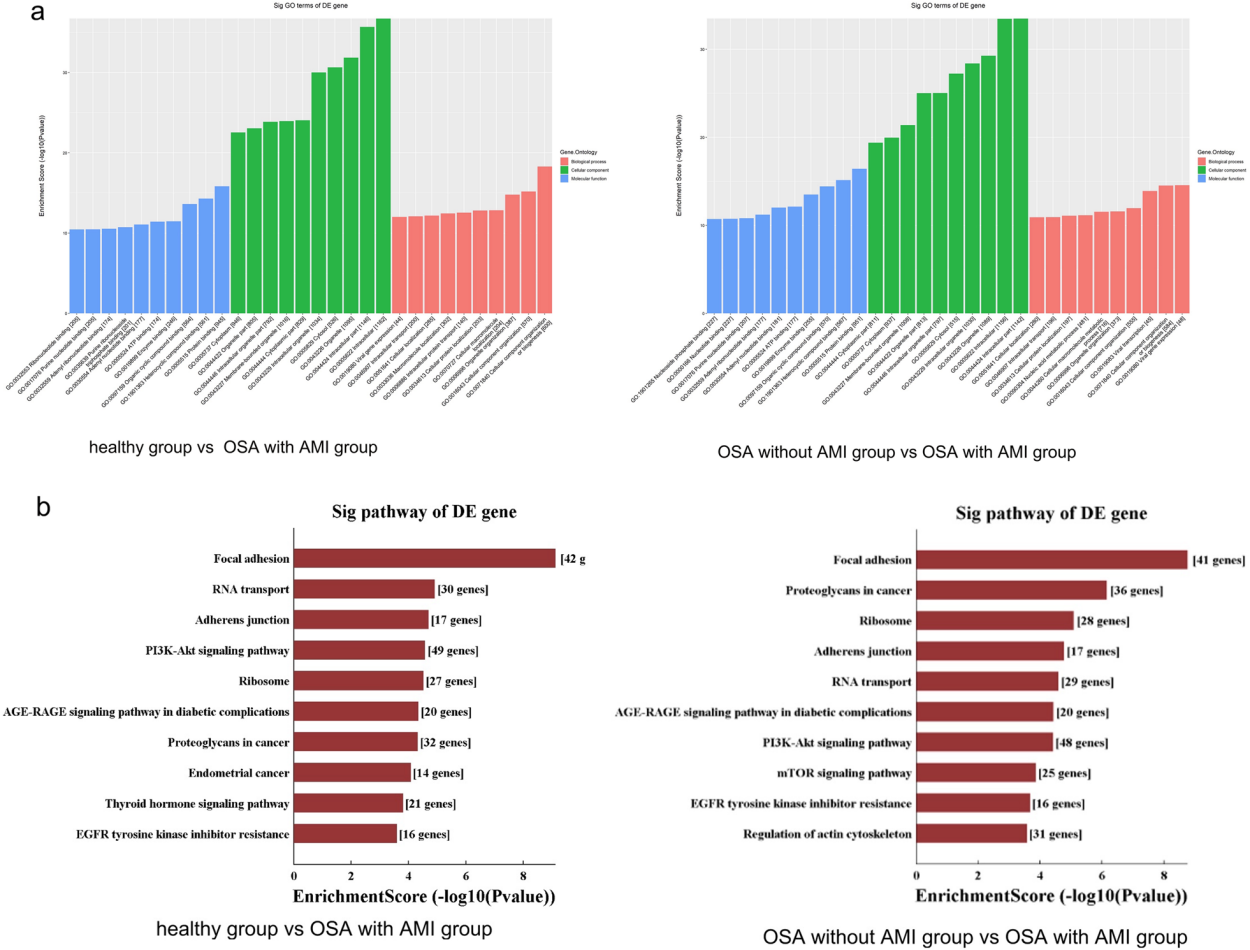
GO and KEGG pathway analysis of dysregulatedcircRNA linear transcripts. **a** GO annotation of the OSA with AMI, versus healthy group and OSA with AMI versus OSA without AMI groups via the top 10 enrichment scores in the BP, CC and MF domains. **b** KEGG pathway analysis of the OSA with AMI, versus healthy group and OSA with AMI versus OSA without AMI groups via the top 10 enrichment scores

GO function analysis was used to speculate the function of circRNAs, which covers three classifications: biological process (BP), cell component (CC) and molecular function (MF). For healthy group versus OSA with AMI group and OSA without AMI group versus OSA with AMI group, the top 10 GO terms of the three aspects BP, MF and CC were almost similar. The results of GO function analysis in comparision of above two groups were as follow: significant GO biological process showed that differentially dysregulated circRNAs were mainly associated with cellular component organization or biogenesis, cellular component organization, organelle organization. GO analysis of cell component showed dysregulated circRNAs were mainly focused on Intracellular, Intracellular part, Organelle, Cytosol. For molecular function, dysregulated circRNAs were mainly concentrated on Binding, Protein binding, Catalytic activity, Organic cyclic compound binding, Heterocyclic compound binding. However, for heathy group versus OSA without AMI, the results of GO showed that the dysregulated circRNAs were rare (Fig. 3a).

### Pathway analysis

Pathway analysis is a functional analysis mapping genes to KEGG pathways, which explore the pathways influenced by the change of circRNAs in serum exosomes. For healthy group versus OSA with AMI group and OSA without AMI group versus OSA with AMI group, “Focal adhesion”, “RNA transport”, “Adherens junction” and “Ribosome” were most enriched in the upregulated genes, and “Ubiquitin mediated proteolysis”, “Protein processing in endoplasmic reticulum”, “Dopaminergic synapse”, “Thyroid hormone signaling pathway” and “Sphingolipid signaling pathway” were in the downregulated genes. Most of dysregulated genes were associated with myocardial injury. And there was almost not enriched pathway for healthy group versus OSA without AMI (Fig. 3b).

### Validation of the accuracy of circRNA-seq data by qRT-PCR

**Fig. 4:**
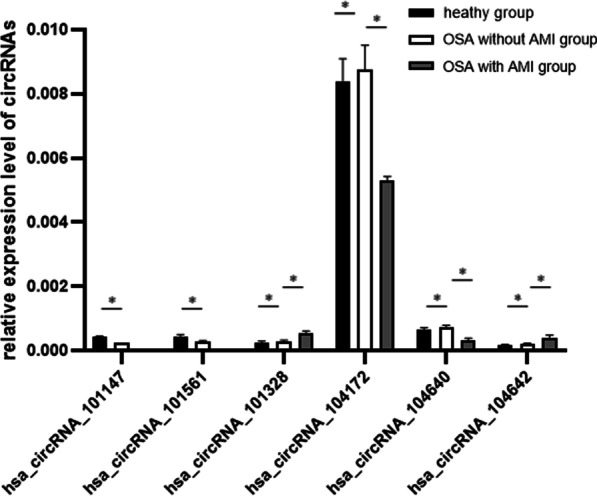
Validation of selected circRNAs, miRNAs and mRNAs by qRT-PCR. 6 circRNAs were significantly amplified by qRT-PCR and consistent with the microarray results *p < 0.05

Based on the significant difference and raw signal intensity of expression, we validated six differentially expressed circRNAs (circRNA_101147, circRNA_101561, circRNA_101328, circRNA_104172, circRNA_104640, circRNA_104642) by qRT-PCR in the same RNA samples used for the microarray analyses. The results of qRT-PCR were in agreement with microarray findings (Fig. 4).

### Identification of circRNA-miRNA network

**Fig. 5:**
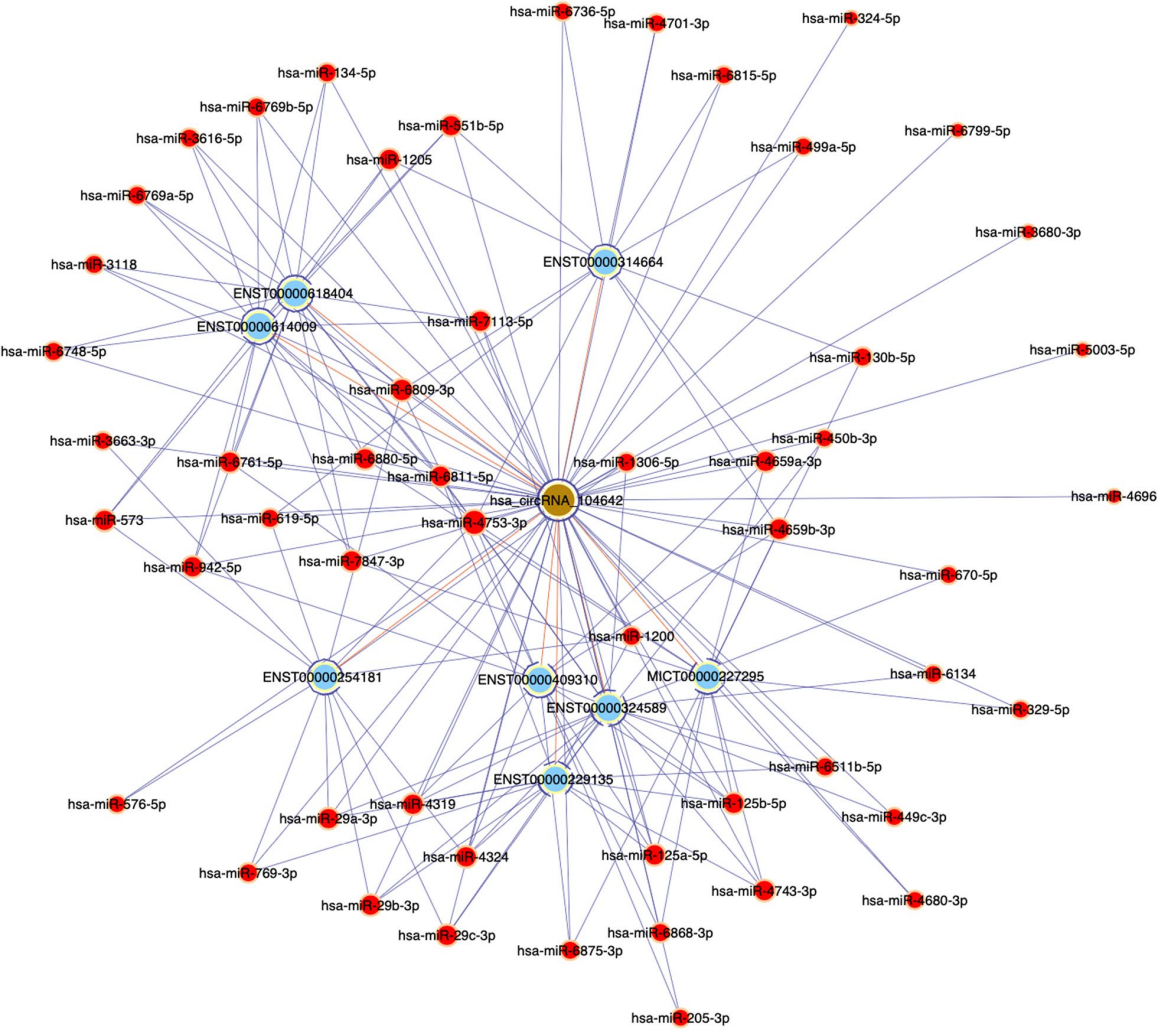
circRNA-miRNA-Mrna network analysis showed circRNA_104640 was observed to act as sponge for 58 miRNAs and 8 Mrna

Bioinformatic analysis revealed that some circRNAs could act as miRNA sponge, RNA binding protein sponge and translational regulator. The networks provided an important reference value to analyze the interactions of circRNAs and miRNAs. We construct circRNA-miRNA-mRNA networks by Cytoscape software. And we found that one circRNA may regulate one or several miRNA/miRNAs in different ways. For example, circRNA_104640 was observed to act as sponge for 58 miRNAs and 8 mRNA according to the analysis of circRNA-miRNA network. It hence appears that there is a complicated circRNA-miRNA-mRNA pathways involved in the pathogenesis of OSA with AMI (Fig. 5).

**Fig. 6:**
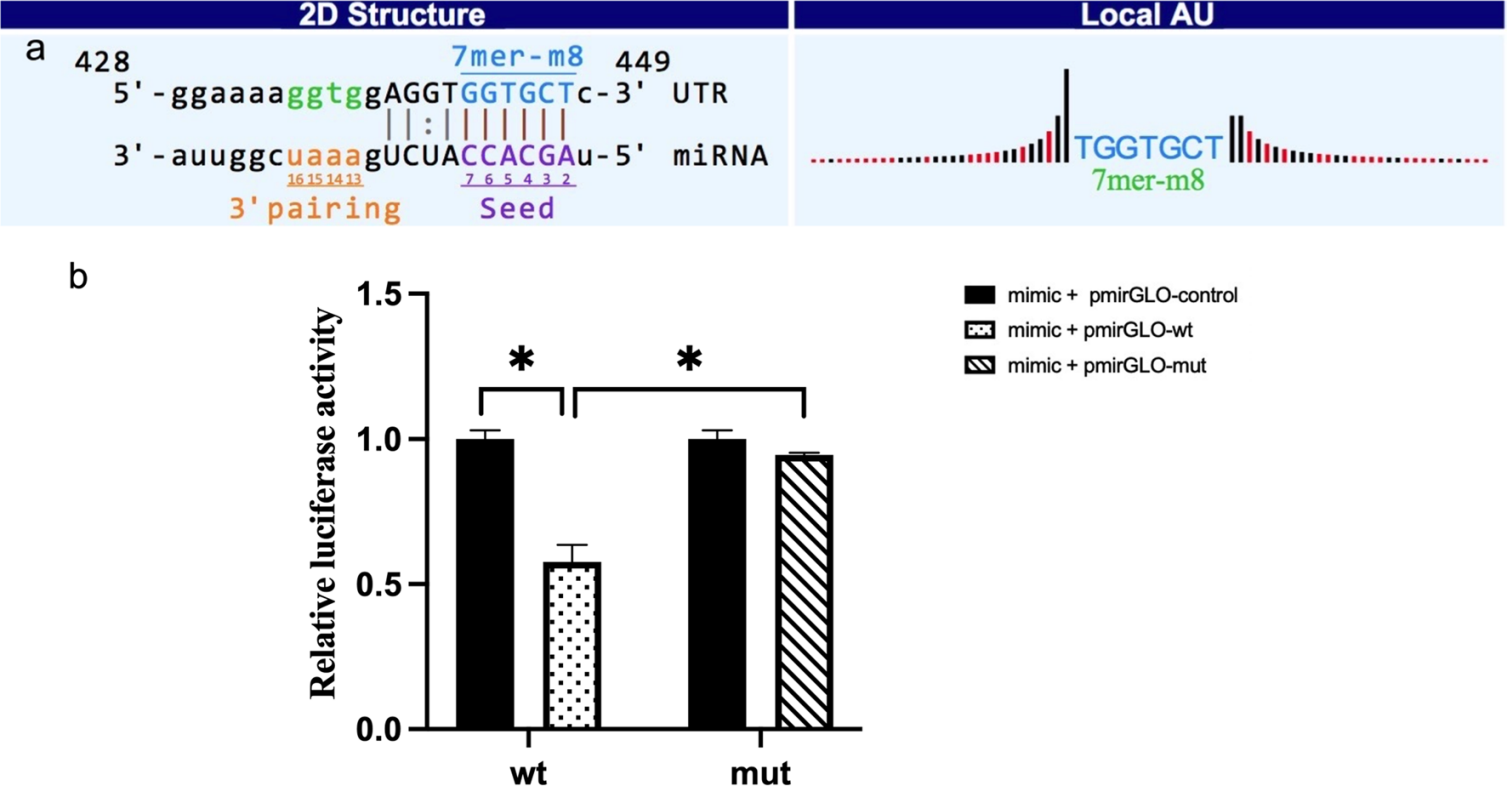
miR-29a-3p acted as a miRNA sponge for hsa_circRNA_104642. **a** Predicted binding site of miR-29a-3p in hsa_circRNA_104642. **b** Dual-luciferase reporter assay showed that hsa_circRNA_104642 mimics significantly repressed the luciferase activity of miR-29a-3p in 293 T cells *p < 0.05

Dual luciferase reporter assay was conducted to confirm whether circRNA_104642 directly targeted miR-29a-3p, the results showed that the activity of luciferase reporter vector carrying the circRNA_104642 3’UTR-WT sequence could be significantly decreased by miR-29a-3p mimics compared with control groups (Fig. 6).

## Discussions

A number of scholars have demonstrated that OSA was associated with increased risk of cardiovascular diseases including AMI, hypertension, cardiac arrhythmias. However, the pathophysiological mechanisms remain poorly understood. It is rendered that circRNAs not only played an important role in the development of cardiovascular disease, but have also been considered as potential biomarkers which served as intracellular signalling molecules for cardiovascular physiology and disease. Therefore, we explored the profile of serum exosomal circRNAs in three group patients (healthy group, OSAHS without AMI group, and OSAHS with AMI group).

Compared with RNA-seq technology, Arraystar Circular RNA Microarray has advantages on quantification of low expressed transcripts, algorithms, building databases, etc. [21, 22]. Therefore, we chose Arraystar Circular RNA Microarray to analyze differentially expressed circRNAs in serum exosomes. And it was found that the expression of serum exosomal circRNAs was the mostly abundant in OSA with AMI patients. Then we verified six differentially expressed circRNAs including hsa_circRNA_101147, hsa_circRNA_101561, hsa_circRNA_101328, hsa_circRNA_104172, hsa_circRNA_104640, hsa_circRNA_104642 according to the significant difference and raw signal intensity of expression. The qRT-PCR results coincided with circRNA microarray analyses and most differentially expressed circRNAs have not been studied yet.

CircRNAs, one class of non-coding RNA, have been suggested to affect the expression of linear mRNA from their host genes and be expected to play a crucial role in cardiovascular diseases. Previously, we have also found that a cluster of dysregulation miRNAs was involved in the development of OSA-associated cardiovascular damage [23]. Thus, we constructed the circRNA-miRNA network to search the underlying relation of the six validated circRNA and adjacent coding gene, which might supply novel perspective for the mechanism of AMI in OSAHS. Our study predicted that circRNA_104642 might function as a decoy of miR-29a-3p and the dual luciferase reporter assay validated our results. The study of Zhang [24] showed that miR-29a-3p significantly decreased in the hearts of cardiac ischemia reperfusion (CIR) injury mouse models and seem contribute to CIR injury-related apoptosis mainly by targeting Bax. Thus, we assumed that circRNA_104642 has a proinflammatory function in fulminant myocarditis by sponging miR-29a-3p to control Bax expression. However, the network of circRNA_104642/miR-29a-3p/Bax in OSA with myocardial injury needs further investigation.

GO and KEGG pathway enrichment analyses were conducted to functionally annotate parental genes of differentially expressed circRNAs in the development of OSAHS with AMI. GO analysis related to cell metabolism and regulation. KEGG pathways for dysregulated genes were enriched in several biological pathways including Focal adhesion, RNA transport, Adherens junction, Ribosome, Ubiquitin mediated proteolysis, Protein processing in endoplasmic reticulum, Dopaminergic synapse. These pathways all contribute to myocardial injury. Therefore, our findings revealed that the change in circRNA expression might contribute to AMI in OSA patients via these pathways.

Several limitations of this study should be mentioned when interpreting the results. First, the number of samples in each group was too small to assess the statistical significance. Further studies should be considered with a larger sample size to validate the function of verified six differentially expressed circRNAs. Second, differentially expressed circRNAs were screened using a bioinformatic method, which should be confirmed by using the luciferase reporter assay and require support from further in vitro and in vivo studies. Thirdly, we did not detect the expression of miRNAs using the same sample to further verify the role of predicted miRNAs in OSA with AMI patients. It will need a large sample size to confirm the expression of some circRNAs and miRNAs in patients with OSA and AMI in the future.

## Conclusions

In summary, our results were the first to provide the expression profile of serum exosomal circRNAs in OSA patients with AMI, which might play a crucial role in the development of myocardial injury in OSA. Moreover, we confirmed the expression of six serum exosomal circRNAs by using qRT-PCR and found circRNA_104642 may be used for the differential diagnosis of AMI in OSA patients. Pathway analysis exhibit a global view of the functions of these differentially expressed circRNAs. These circRNAs may serve as the possible therapy targets of myocardial injury in OSA patients.

## Data Availability

The datasets generated during the current study are available in the [GEO data(GSE197137)] repository.

## Abbreviations

circRNAs: Circular RNAs
OSA: Obstructive sleep apnea
AMI: Acute myocardial infarction
STEMI: ST-segment elevation myocardial infarction
DEGs: Differently expressed genes
AHI: Apnea–hypopnea index
LaSO_2_: Lowest oxygen saturation
GO: Gene ontology
KEGG: Kyoto encyclopedia of genes and genomes
qRT-PCR: Quantitative real-time PCR
BP: Biological process
CC: Cell component
MF: Molecular function
